# Young people and their engagement with health-related social media: new perspectives

**DOI:** 10.1080/13573322.2017.1423464

**Published:** 2018-01-25

**Authors:** Victoria A. Goodyear, Kathleen M. Armour, Hannah Wood

**Affiliations:** a School of Sport, Exercise and Rehabilitation Sciences, University of Birmingham, Birmingham, UK; b Pro-Vice- Chancellor-Education, University of Birmingham, Birmingham, UK

**Keywords:** Health, public pedagogies, digital media, Instagram, Snapchat, Youtube, cultural genres, physical activity, diet, body image

## Abstract

Young people are increasingly turning to social media for health-related information in areas such as physical activity, diet/nutrition and body image. Yet, there are few robust empirical accounts of the content and form of the health-related material young people access and attend to, or the health-related content they create and share. Furthermore, there is little guidance from research or policy on young people's engagement with health-related social media. This leaves many relevant adults ill-equipped to protect young people from the negative influences of social media and to optimize the potential of social media as a medium for health promotion. This article presents new evidence on young people's engagement with social media and the influences they report on their health-related behaviors. The research was undertaken with 1296 young people (age 13–18) using a participatory mixed methods design. Initially, a public pedagogy [Giroux, 2004. Public pedagogy and the politics of neo-liberalism: Making the political more pedagogical. *Policy Futures in Education*, *2*, 494–503] theoretical framework was used to guide data analysis but this was found to be limiting. An adapted framework was developed, therefore, drawing on Miller et al. [2016. *How the world changed social media*. London: UCL Press] and Lomborg [2011. Social media as communicative genres. *Journal of Media and Communication Research*, *51*, 55–71] to account for the unique ways in which pedagogy operates in a social media context. Young people accessed and used a range of health-related information on body transformations, diet/nutritional supplements or recipes and workouts/exercises, albeit in different ways. Moreover, young people identified five forms of content that influenced their understandings and behaviours: (i) automatically sourced content; (ii) suggested or recommended content; (iii) peer content; (iv) likes; (v) reputable content. The findings also suggest that relevant adults can reduce risk and realize more of the positive impacts of social media for young people by focusing on content, and the ways in which content is shaped in the interplay between interactive functionalities of social media (e.g. likes and followers) and young people's social uses of social media (e.g. friends, information).

International evidence suggests that young people are increasingly turning to social media for health-related information, in areas such as, physical activity, diet/nutrition and body image (Swist, Collin, McCormack, & Third, 2015; Wartella, Rideout, Montague, Beaudoin-Ryan, & Lauricella, 2016). Yet, there are few robust empirical accounts on the types of health-related information young people access and attend to, nor the health-related content that they create and share on social media (Haussmann, Touloumtzis, White, Colbert, & Golding, 2017; Holmberg, Chaplin, Hillman, & Berg, 2016). Many relevant adults who are invested in young people's health and wellbeing (including teachers, parents/guardians, health professionals/practitioners, policy makers and researchers) are aware that young people are prolific users of social media, but they are uncertain about how this behavior influences young people's health-related knowledge and behaviors (Frith, 2017; Haussmann et al., 2017). As a result, we have a poor understanding of how to position young people in the social media-health nexus, and there is a tendency to focus mainly on risk and negative outcomes of use (Shaw, Mitchell, Welch, & Williamson, 2015; Third, Bellerose, Oliveira, Lala, & Theakstone, 2017). No robust guidance on young people's health-related uses of social media is available for researchers, practitioners or clinicians (Haussmann et al., 2017; Third et al., 2017) and there is also little mention in school/childcare guidelines in current European, UK and US policy (Frith, 2017; Wartella et al., 2016). There is, therefore, a gap in this research and policy space that leaves many relevant adults ill-equipped to support young people in their engagement with social media. The support that these adults could provide is twofold: to help young people deal with the risks attached to the vast amounts of widely available unsolicited and unregulated health-related digital material (Livingstone, Mascheroni, & Staksrud, 2017; Third et al., 2017); and to optimize the potential for social media to be a powerful and positive educational tool to inform young people's health-related understandings and behaviors (Shaw et al., 2015; Third et al., 2017).

Understanding the health-related opportunities and issues generated by social media from the perspective of young people is an essential starting point for developing new and more effective health promotion interventions (Mascheroni, Jorge, & Farrugia, 2014; Third et al., 2017). This article presents new evidence on the ways in which young people engage with health-related social media and the influences they report on their health in the specific areas of physical activity, diet/nutrition and body image. The research questions were: (i) What health-related information is/isn't accessed by young people through social media; (ii) What health-related information do young people attend to and use to inform their health-related behaviors.

## Young people, social media, and health

It has been reported in numerous international and socio-economic contexts that young people have the highest rates of social media use of any age group, and that they spend significant proportions of their time ‘on’ social media (see Third et al., 2017). Young people are, therefore, avid users and drivers of this contemporary, participatory and user-driven online culture. To some extent, young people can be understood as highly skilled and knowledgeable about social media.

Given the significance of social media in young people's lives, it is a powerful space in which to reach young people and—potentially—to impact on their health in both positive and negative ways (Haussmann et al., 2017; Third et al., 2017). In the case of adults, reported health-related benefits include: increased interaction; more available, shared and tailored information; increased accessibility to health information; peer/social/emotional support; and health surveillance (see Shaw et al., 2015). Yet, there is limited evidence on how these benefits are realized in practice, and even less evidence in relation to young people (Haussmann et al., 2017; Shaw et al., 2015).

Public discourse on young people and their uses of social media tends to focus almost exclusively on risk (Livingstone et al., 2017; Shaw et al., 2015). There is abundant literature on the negative impacts of social media in areas including: reduced physical and psychological health due to sedentary lifestyle, loss of sleep and associated cognitive impairment; risks for mental health, such as anxiety, stress and body dissatisfaction; and impact on cognitions such as negative self-perception, bullying and social isolation (Frith, 2017; Shaw et al., 2015). This pervasive risk narrative means that adults may be unaware that there is also the potential for social media to act as a powerful health promotion tool (Haussmann et al., 2017).

In order to minimize the risk-related impacts of social media, highly protection-orientated approaches tend to be adopted, that seek to limit and control young people's social media use (Third et al., 2017). Young people, however, have very different views and experiences of social media when compared to adults. In existing research, young people have reported benefits in the areas of: learning, socialization, increased access to information, greater levels of social and emotional support, and creativity (Frith, 2017; Swist et al., 2015; Third et al., 2017). There would appear, therefore, to be a large gap between the ways in which young people and adults understand social media. In order to realize more of the positive impacts, it is important to better understand these gaps and to find new ways to acknowledge young people's social media knowledge, skills and experiences.

To date, our understanding of young people and social media has been methodologically and theoretically constrained. Most studies have treated young people as passive, with evidence based largely on parent/guardian and teacher perspectives and/or conclusions drawn from content analysis and observational methods (James, 2014; Mascheroni et al., 2014). In contrast, evidence from large-scale research into digital media identifies what can be gained when young people are positioned as active agents in the research; for example, insights can be generated into young people's discourses about risk, how they negotiate and navigate digital environments, and the spaces and cultures to which adults often don't belong or understand (James, 2014; Mascheroni et al., 2014; Third et al., 2017). Theoretically, most studies have been grounded in psychology and lack insights into the pedagogical processes of digital media (Rich & Miah, 2014). This paper therefore makes an original contribution to the fields of health and education by examining young people's experiences of health-related social media from their perspectives and in new ways that reveal the importance of pedagogical processes.

## Public pedagogies

Often grounded in the works of Giroux (2004), public pedagogies is a concept that acknowledges learning as an experience influenced by culture, that is not confined to formal education spaces (Burdick & Sandlin, 2013). The medium of social media is a contemporary example of the concept of public pedagogies (Andersson & Olson, 2014), which is identified as a public place where individuals go to learn and where learning occurs indirectly through connections with others (Andersson & Öhman, 2017; Reid, 2010).

According to Giroux (2004) pedagogy is not a technique or *a priori* set of methods, but a political and moral practice. As a political practice, pedagogy illuminates the relationship between power, knowledge and ideology. As a moral practice, pedagogy recognizes that what the media teaches cannot be abstract from what it means to invest in public life; for example, to locate oneself in a public discourse. Accordingly, Giroux (2004) defined public pedagogy as the regulatory and emancipatory relationship between culture, power and politics that occurs in a democratically configured social space. In the social space, the cultural field ‘plays a central role in producing narratives, metaphors, and images that exercise a powerful pedagogical force over how people think of themselves and their relationships to others’ (Giroux, 2004, p. 62). In turn, Giroux (2004, p. 498) claimed that ‘knowledge and desire are inextricably connected to the modes of pedagogical address’. For example, films are a mode of pedagogical address (Giroux, 2002). Through particular representations, in text, images, sounds, gestures or dialogue, films operate pedagogically (i.e. politically and morally) through the ‘common sense assumptions that inform them, the affective investments they mobilize, as well as the absences and exclusions that limit the range of meanings and information available’ (Giroux, 2002, p. 539).

Although Giroux's work has impacted significantly on the field of public pedagogies (Burdick & Sandlin, 2013), connecting Giroux's conceptualization of public pedagogies to contemporary digital media is problematic. For us, as for others, it was challenging to apply Giroux's concept that was grounded in a passive media context to a dynamic social media context (e.g. Andersson & Olson, 2014; Andersson & Öhman, 2017; Reid, 2010). While social media can be interpreted as a mode of public pedagogy (Andersson & Öhman, 2017), there are diverse modes of social media (e.g. SnapChat or Instagram) that include varied and multi-dimensional interactive functionalities. Further, although social media can be seen as a democratically configured space, autonomy and control are effected reflexively through the simultaneous processes of liberation and discipline enacted by the user and the interactive functionalities (Andersson & Olson, 2014; Papacharissi, 2014). Thus, an analytical framing of pedagogy that operates through power seems to neglect the role of users shaping content and educational experiences (Andersson & Olson, 2014; Papacharissi, 2014).

While advances to the concept of public pedagogies have been made through a focus on the user (see Burdick & Sandlin, 2013), pedagogy is often used in ways that are indistinguishable from socialization theories (Savage, 2014). Well-established definitions of pedagogy, that place learning and learners’ needs at their core, and focus on the alignment of context and teaching practices (Armour & Chambers, 2014; Casey, Goodyear, & Armour, 2017) are also problematic to apply in a social media context. These definitions of pedagogy do not account for the absence of a teacher, instructor or facilitator in public and social media environments (Andersson & Olson, 2014; Savage, 2014). As a result, we required a new framing of pedagogy that accounted for the dynamic relationship between the user (i.e. young people) and the interactive functionalities of social media; one that could reflect both democracy and control in the construction of content and educational experiences. The anthropological work of Miller et al. (2016) was helpful in better understanding the operation of pedagogies in a social media context.

Miller et al. (2016) argue that understandings of social media in an educational framework must be centered on content, given that content migrates through social media platforms and is an enduring entity in the dynamism of online/digital media. A focus on content is also important because specific digital content is able to persist across different cultures and amongst varied social groups, even though the meanings assigned to that content may differ (Miller et al., 2016). This is not to argue that content is passive, but rather it is something that users do and is actively constructed through different cultural genres (i.e. styles of use) (Miller et al., 2016). Cultural genres are grounded in the work of Goffman (1959), who argued that communication and sociality take place within cultural genres. In the context of social media, cultural genres provide analytical tools on the level of communicative practice (Lomborg, 2011). Genres are ‘constituted at the interplay between different interactive functionalities configured in software and the distinctly social purposes that users orient to in their communicative practice’ (Lomborg, 2011, p. 57). Content, therefore, places the focus of analysis firmly on the active user and their interactions with content (Miller et al., 2016). This theoretical lens aligns well with the focus on young people's perspectives that underpinned our research.

To understand young people's experiences of health-related social media, Miller et al. (2016) and Lomborg (2011) suggest that we should consider pedagogy in the context of social media a different way. This work directs the focus towards content, and both the dynamic interaction with and generation of content as a key analytical lens. At the same time, elements of Giroux's conceptualization of public pedagogies remained relevant to the data analysis process; in particular, the importance of understanding the powerful role of common sense assumptions, affect, and the absences and exclusions of information. In the discussion, we explore these issues further, illustrating how the data analysis process led us to rethink the concept of public pedagogy in the social media context.

## Methods

A participatory and iterative research design was adopted to generate rich, in-depth and detailed insights into young people's experiences of social media. Young people were active participants in the research and a multi-method approach was used to engage with multiple and varying sample sizes of young people at different stages.

### Ethics

A culturally responsive relational and reflexive approach to ethics was adopted (Sparkes & Smith, 2014). This approach ensures that ethical decision-making is contextualized within the digital cultures and contexts inhabited by young people (Goodyear, 2017). Following this approach, care for participants involved creating and using data collection methods that ensured participant safety, privacy and dignity, and that promoted participant autonomy. University ethical approval was granted and informed consent or assent were obtained from participants. Legal conditions of social media were adhered to by using university accounts and devices, and ensuring participants were age 13 or over.

### Context and participants

Data collection took place with 1296 young people (age 13–18) from 10 UK schools located in the West Midlands and the South of England. Of the 10 schools, 2 were private, 3 were state, and 5 were academies, 2 of which were faith schools.^1^ The schools were located in diverse socio-demographic areas and included students from a range of ethnic backgrounds, with just under a third of students across these schools speaking English as a second language.

### Data collection

Data collection was framed by an initial focus on health-related social media in the areas of physical activity, diet/nutrition, body image and sleep. A conceptually narrow focus on health-related content was taken to provide clarity for participants, and depth in the data on the types of material accessed and attended to, and the conditions of young people's experiences.

Data collection took place over 10 months and across three iterative phases where data generated from each phase informed data collection techniques in the next phase. An overview of methods used in each of the three phases is provided in Table 1.

**Table 1.** Data collection methods.

Method	Description of the Method
Participatory Class Activities (*n *= 236, age = 13–15, *m* = 101, *f *= 135)	Young people worked in groups of 4–5 members, that were self-selected to support engagement in discussions. Each group completed a series of activities presented to them in an iBook. Data were acquired from 2 activities. *Class Questionnaires (n = 236; m = 101, f = 135, age = 13–15):* Each group watched a 2-minute video created by the researchers on current statistics of health-related social media use. Young people then individually completed a questionnaire, in the form of a leaflet. The class questionnaire was closed/open ended and was composed of 5 questions related to their uses of social media. Mean percentages were calculated for closed questions. Open-ended questions were categorized inductively and mean percentages were then calculated in relation to each category. *Digital Pin Board (n = 53; m = 22, f = 29, mixed = 2 age = 13–15):* A Pinterest digital pinboard was co-constructed with young people (phase 1) on different types of health-related images and videos available on social media. 55 images/videos were grouped into 11 categories: female body image, workouts, clean eating, sleep/mental wellbeing, motivation, physical activity, campaigns, male body image, governing body advice, commercial brands and celebrities. Inter- and intra- reliability tests were completed to confirm categorization. A level of 85% was deemed appropriate (Van der Mars, 1989) and reached before the pinboards were used. In phase 2, each group was asked to either keep or delete the images. In turn, the pinboards provided data on the categories of health-related material young people attend to and would use. Mean percentage were calculated on the categories kept across all groups.
Interviews (*n* = 84; age = 13–15, *m* = 35 *f* = 49)	19 interviews (20–40mins) were conducted in the same groups from the class activities. Two groups per class were interviewed (where possible). Groups were selected on the basis of offering a balanced sample on the health-related material young people access/attend to across the 10 schools and gender. Elicitation techniques were firstly used to encourage young people to discuss their pinboard. Semi-structured questions were then used to understand young people's experiences of social media, and were common across all groups. The interviews complemented data obtained from the class activities
Online Survey (*n *= 1016, age= 13–16, *m* = 334, *f* = 676)	The online survey was constructed based on data from phase 2 and was administered through an online platform. The online survey differed to the class questionnaire by containing 33 closed multiple choice questions organized into 8 sections: Background (gender/age/school); How do you use social media?; What do you look at on social media?; What do you do when you see a post?; What do you change if you see a post?; Do you post anything about your health?; Is social media good for your health? Mean percentages were calculated for each question.

Phase 1 involved piloting and co-constructing participatory class activities with a group of 10 young people (age 16–18; *m *= 6, *f *= 4) who were not involved phases 2–3. This process occurred over 2 months and was designed to ensure that data collection techniques appropriately reflected young people's uses and experiences of social media, and that the pre-selected areas of health-related focus were relevant to the material young people access.

Phase 2 involved generating data from 12 school classes of young people (*n *= 236; *m* = 101, *f* = 135) through participatory class activities and interviews (Table 1). The classes were single sex (*m* = 3, *f *= 5) or mixed gender (*n* = 4) and data collection took place in physical education (*n *= 10) or citizenship lessons (*n *= 2). Initially, participatory class activities were led by a researcher and took place in a one-hour lesson. Using the data generated, 19 focus group interviews (*n* = 84; *m* = 35, *f* = 49) were then conducted in a subsequent lesson (see Table 1). In combination, the class activities and interviews provided a rich data set on the health-related information young people access and attend to on social media.

Phase 3 involved generating data from an online survey that was designed to take account of the data from phase 2. The online survey was piloted initially (*n* = 34; age 13–16; *m *= 18; *f *= 16) and was then completed by 1016 young people (*m* = 334; *f* = 676; age, 13–16) from the same 10 schools as phase 2, but from different classes (see Table 1). The online survey acted to validate the data from phase 2 with a wider sample of participants.

### Analysis

Analysis was ongoing and iterative throughout the study to facilitate data collection across the three phases. At the end of the study, analysis then took place in two overarching phases and is outlined in Tables 2 and 3.

**Table 2.** An illustration of the process of coding in phase 1 of analysis.

Accessed sample codes	Attended to sample codes	Representative Categories	Knowledge or Behaviour
Transformation 2 pictures Mirror Smoothies Tea Clean Eating Muscles Weights HIT Motivation	Transformation 2 pictures Mirror Smoothies Tea Clean Eating Muscles Weights HIT Motivation	Body Image (transformations, 2 pictures, mirror) Diet/Nutrition (Smoothies, Tea, Clean Eating) Physical Activity (muscles, weights, HIT)	Slim Thick Drink Tea Protein Buff Fitter Healthier Pressure Self-esteem Negative perception Jealous

**Table 3.** An illustration of the process of coding in phase 2 of analysis.

Interactive Functionalities of Social Media	Social purposes that orientate users	Genres	Content/Themes	Pedagogical Operations
Likes Followers Suggested Recommended Automatic Accounts Reach Images Videos Filters Selfies Search Search and Explore	Attention Search Following Friends Celebrities/consumerism Endorsement Information Credibility Shortcuts Networks	Intimacy Likes, followers, friends, following, automatic, Search and Explore, networks Information Seeking Videos, automatic, search, information, suggested, recommended Performativity Selfies, attention, images, filters, endorsement Affirmation Likes, information, credibility, friends Credibility Followers, reach, credibility, images, videos, information	Automatically Sourced Content Suggested/ Recommended Content Peer Content Likes Reputable Content	Assumptions Affect Limits Affect Affect Assumptions Affect Assumptions Affect Limits

Phase 1 (Table 2) involved organising the data sets into the types of health-related information that were accessed and attended to. A refined approach was then adopted to identify the types of knowledge and behaviours that were associated with this information.

Phase 2 (Table 3) was grounded in pedagogy as we had originally defined it and aimed to examine how young people's knowledge and behaviours were influenced, or not, by the health-related information accessed/attended to. The organized data sets (as per phase 1) were considered in order to identify different forms of content. Consistent with the understanding that content is shaped by the user and their interactions (Miller et al., 2016), the data sets were coded by: (i) interactive functionalities and (ii) social purposes (Lomborg, 2011). The interplay between the interactive and the social was then investigated and this process identified 5 genres and illustrated how the technical and social features worked together to construct content. For example, the interplay between (i) likes, followers, Search and Explore, automatic (interactive), and (ii) friends and following (social), was associated with intimacy, and automatically sourced content. The categories of content were then coded by: (i) common sense assumptions, (ii) affect, and (iii) the absences or exclusions of information. This process aimed to interpret how the content influenced understandings and behaviours and was also informed by Giroux’s (2002) example of the ways in which static media—such as films—operate pedagogically. The data indicated that how the content operated pedagogically was associated with the same social and interactive features that constructed the content. The social and interactive construction of content was, therefore, central to understandings of pedagogy in a social media context, and the five categories of content were selected as the main themes.

In all stages of the analysis a deliberative strategy (see Goodyear, Kerner, & Quennerstedt, 2017) was used. The goal of the deliberation was to form a collective agreement where all co-authors were given the possibility to make judgments in relation to different alternatives, views and arguments. The researchers independently constructed codes and these became the basis for deliberation. The authors’ different backgrounds and experiences together with the respectful endeavor to clarify points of agreement/disagreement are indictors of quality in a deliberative process.

### Validity

A relativist approach to validity aimed to extend the robustness of traditional measures of quality and validity (Smith & McGannon, 2017). In this study, the list of characterizing traits that guided validity (Smith & McGannon, 2017) included the following criteria: the worthiness of the topic; the significant contribution of the work; width—meaning the comprehensiveness of evidence from a wide sample of participants from diverse contexts, as well as multiple data and in-depth collection methods; credibility, through the iterative phased design and the co-constructed methods with young people, alongside the analytical process between the researchers about the fairness, appropriateness and believability of the interpretations offered; and coherence, in terms of how this study hangs together in terms of purpose, theory, methods and results.

## Results

The online survey revealed that the majority (53%) of young people used social media actively to look for (i.e. access) health-related material. The class questionnaire data added further evidence on the types of health-related information young people accessed and this was related to physical activity (60%), diet/nutrition (55%) and body image (8%). The common types of information young people see were related primarily to body changes or transformations as a result of physical activity workouts and/or diet/nutritional supplements:

Get the body you want and it is like before and after (Interview: school 2, male)

People who try and work out and then there is a picture of two pictures of themselves (Interview: school 2, female).

Whether young people actively seek out health-related material—or not—there was consensus in the online survey data that many would also swipe past (i.e. suggesting they are disregarding) material related to physical activity (57%), diet/nutrition (61%) and body image (57%). These data suggest that young people do not attend to (i.e. use) all of the health-related information they access from social media. Yet, even though they swiped past the information, it would appear that the material still impacted on their health-related behaviours given that 46% of young people reported they had changed their health-related behaviours because of something seen on social media (Online Survey). Most young people reported that the health-related material they had seen on social media impacted positively on their health-related behaviours (43%), with fewer reporting negative impacts (24%) (Online Survey).

The data revealed five forms of content that young people identified as influences on their health-related understandings and behaviours, albeit in different ways. It would appear that the content was influential because it operated through affect, promoted common sense assumptions and limited and/or excluded young people's access to a wider range of health-related information.

### Automatically sourced content

Automatically sourced content refers to the health-related information that social media sites pre-select and promote to young people. For example, Instagram pre-selects categories of content (e.g. recipes, animals) that users see on the ‘search and explore’^2^ feature, based on: a user's likes, who that user follows and their followers’ likes, and automatically sourced accounts. Within the search and explore feature, young people reported that they saw health-related information posted by people they didn't follow and/or commercial companies (i.e. automatically sourced accounts):

When you go on the explorer bit where there are loads of random ones that you don't follow, then you get loads of health ones and before and after pictures. (Interview; school 5, female)

The young people clearly attended to the pre-selected health-related information as evidenced by the ways in which they could describe the information presented. A notable example is FitTea^3^ and ‘Slim Tea’.^4^ The advertisements for these types of tea were based on slender women drinking the tea with images strongly encouraging consumption:

Female 1: They take a picture of their body and they are holding a bottle … 

Female 2: That's what's her name from ‘Cash me outside’. She said ‘I’m not asking you to use Slim Tea, I’m telling you to use Slim Tea … .

Interviewer: What kind of body shape do they have?

Female 3: Toned

Female 1: They are skinny, they are toned

Female 3: Slim thick (Interview; school 10)

The young people associated FitTea and/or Slim Tea with health: ‘it's some tea that makes you healthy … it's like protein in a tea’ (Interview; school 5, female); ‘it's like a detox’, ‘it's like green tea’ and ‘it's for a flat tummy’ (Interview; school 10, female). According to the young people, FitTea was ‘really popular’ (Interview; school 5, female). While none of the young people suggested that they had used FitTea, they were aware of other people who were drinking FitTea or other similar teas: ‘loads of people are drinking green tea now and loads of different healthy teas’ (Interview; school 5, female); ‘they have started taking things that are bad for the body and then they like think that it might be doing something good, but it's not really’ (Interview; school 4, male).

The example of FitTea and Slim Tea illustrate how health-related information reaches young people through automatically sourced content. This type of content was mainly shaped by the social media networks young people chose to participate and the interactive features of likes, followers, and automatically sourced information. Automatically sourced content promoted information that formed a set of common sense assumptions that led some young people to associate FitTea and Slim Tea with health. The existence of FitTea and Slim Tea in young people's social media networks also suggests that automatically sourced content influenced young people through affect, and by mobilizing emotive feelings toward health that were shared extensively within a network. The absence of this material in some young people's networks, and its appearance through search and explore, may explain why some young people did not report accessing or attending to Slim Tea or Fit Tea.

### ‘Suggested’ or ‘recommended’ health-related content

This theme refers to the process whereby young people's ‘searches’ for specific health-related information result in social media sites then promoting vast amount of partially related material to their accounts. Young people searched social media for health-related information that they considered to be relevant to their health. Material considered to be most relevant was related to motivation for exercise (78%), clean eating (68%) and physical activity workouts (67%) (Digital Pinboards). Following a young person's initial search however, different health-related information related to similar topics was ‘suggested’ or ‘recommended’, appearing on these young people's home pages or timelines.

Male 1: I’ll find something by a specific YouTuber, and then I’ll get recommended a bunch of their other videos … 

Male 2: You can get an infinite loop on YouTube. I might just start watching one video and then another interesting video will pop up and you’ll watch that (Interview; school 8)

 

There are so many suggested images, like transformations. Like you see a girl that is really big and then six weeks later she's really slim now (Interview; school 5, female)

Most of the young people understood that much of the ‘suggested’ or ‘recommended’ material was targeted at adults and was inappropriate for their age: ‘It's not really our age, because they’re like in their early 20s and just a bigger age group’ (Interview; school 9, female). Yet many of the young people spent a significant proportion of their time engaging with these videos: ‘I’ll go through a few days just repeatedly watching their videos’ (Interview; school 8, male). Some young people perceived that the information presented in the videos benefited their health given that the material was highly accessible, varied and provided them with short duration and simple solutions to becoming ‘healthier’. Other young people, reported that the quick solutions were not relevant to their bodies and were—anyway—unachievable.

There's easy smoothies that you can make within seconds and usually when it comes to dieting … we’d be like “oh it's going to take time, we’re not going to get there” …  They make it so simple that we can just do it within seconds (Interview; school 3, female)

It's definitely helpful because it's like the way we live, it's like we are always looking down and if we can get the quickest way find out how to be healthy or something and it's on an Instagram page or something, it just makes life easier (Interview; school 8, male)

Sometimes, if I see someone working out on their posts and they say, “I’ve lost this much weight,” it makes you feel like you have to do that. They might have done that in two days, whereas, for me, it might take two weeks to do it. In a way, that might put me down (Interview; school 6, female)

Social media sites ensure that vast amounts of health-related material can reach young people through the process of recommending and suggesting content. Suggested or recommended content was shaped by a young person's search for health-related information (social) and suggested or recommended content (interactive). This type of content limited and/or excluded young people's access to a range of information. Some young people, however, resisted the content, as evidenced by their understanding that most of the health-related material was inappropriate for their age. Yet, due to affect, some young people chose to engage with and use this health-related material. Affect was evident through the content that promoted either: feelings that health-related changes were achievable through, for example, ‘shortcuts’; or negative body comparisons.

### Peer content

This theme refers to the health-related content that young people create and how this material encouraged other young people to engage in body comparisons with their own bodies. 1 in 4 young people reported that they created content on social media related to health (26%) in the areas of physical activity (9%), diet/nutrition (4%), and body image (6%) (Online Survey). This content had a wide reach, given that young people reported seeing vast amounts of health-related content created by people of their own age. The content was often represented in the form of selfies:

People take a picture of the food they are eating (Interview; school 4, male)

I’ve seen some guys our age posting topless pictures if they’re ‘ripped’ or whatever (Interview; school 8, male)

Pictures of their belly saying, ‘working on a diet’ (Interview, school 5, female)

Young people reported that the selfies created by other young people of the same age could act as a form of peer pressure. While there was consensus that the reasons other young people created this content was to ‘seek attention’ (Interview; school 5, female), the content made some young people feel that they should change their health-related behaviors. This type of pressure resulted in young people questioning their bodies and their appearance:

Female 1: I think it puts pressure on you … 

Female 2: When people our age that we know post photos of them and their body … 

Female 1: Saying bad stuff about their figure, when they clearly know that their figure can't be that bad otherwise they wouldn't have posted the picture in the first place.

Female 2: When their figure is better than mine and they’re saying that they’re fat … 

Female 1: Then you think that yours is 10 times worse (Interview; school 1)

 

Peer pressure, proper peer pressure is like a bigger problem than cyber bullying. Because cyber bullying is a lot more noticeable than the peer pressure, because you might just see loads of different things that make you think that you should do something. (Interview; school 1, female)

 

Like be all down about it. Or like ‘I wish my body was like that’ (Interview, school 4, male)

Peer content was shaped by young people themselves creating image-based content, mainly selfies, and this behavior was attributed to the social purpose of attention seeking. Viewing content that was created by peers led some young people to report that they felt peer pressure. This led some to develop negative feelings about their own bodies while others reported wanting to change their appearance and engage with particular health practices. In this sense, peer content had an affective influence.

### Likes

Likes are an interactive functionality, but they were also identified as a form of content due to their interrelation with varying social purposes. The social purposes included the affirmation of: health-related information, a young person's body type and social presence.

Firstly, young people used the number of likes an image or video attracted to affirm whether they should engage with and/or use the health-related information they saw. In turn, likes mobilized common sense assumptions about health:

Interviewer: What's a good amount of likes?

Female 1: 500

Female 2: 100, or like 110 … .

Interviewer: So if a picture of something—an exercise someone was doing—had 100 odd likes, does that show it's good? … 

Female 1: It doesn't appeal to us as much, because it's only 100 likes—so it shows that if other people are not interested, why should we. (Interview; school 3)

Male 1: If it looks interesting or it looks helpful and it's got no likes, you’re just like, ‘No one's life has been improved by this effort … 

Male 2: If it's got a lot of likes, you might try to recreate it … 

Male 1: Oh, people like this. I’ll do that. (Interview; school 8)

Secondly, liking someone's post was a social process that affirmed a young person's body type and had an affective influence on young people's perceptions of their bodies. For example, if a person liked a post made by another young person, this was positioned as an endorsement for the behavior displayed in the post. If the person who liked the post didn't get a like back on their other posts—from the person who made the original post—this acted as a form of judgement on their behavior or body type.

Female 1: The really muscly people, if you were to ever like them or something, they wouldn't like you back because you might be not as skinny as they want you to be and they’ll be obsessed over the weight.

Interviewer: So you think that if you liked it, you would reinforce what they are doing; is that what you mean? … 

Female 1: If I look at your profile and say, “I don't want to know that person,’ but then they say, ‘Oh, yeah I do. Maybe I could help them lose the weight.’ But I not might want to lose the weight. They are predicting that I want to and that I want to be like them, when I might not necessarily want to (Interview; school 5)

Thirdly, likes acted to affirm social presence. Young people described that likes could be used to affirm that you had seen another person's post: ‘[someone] could just be liking it to let you know that they have seen it’ (Interview; school 6, female). In turn, some young people considered likes to be ‘fake’ (Interview; school 6, female) and that material that was liked would make ‘no real difference’ to their health-related behaviors (Interview; school 8, male).

The varying social purposes of likes highlights the diverse ways in which likes operate in young people's social media environments. Likes operated through mobilizing common sense assumptions and affect.

### Reputable content

Reputable content refers to the power of specific social media accounts in framing the types of health-related information young people access and attend to. Social media accounts that young people reported as having the most power were: official organizations (e.g. National Health Service, government, Football Association, Sport England, Youth Sport Trust); celebrities (e.g. singers or actors); sports men and women; and commercial brands (e.g. Nike or Adidas). These types of accounts have a high number of followers and this could be a provide a powerful platform from which to potentially reach and influence young people in both positive and negative ways.

It's better coming from them because they have a platform. So like to us it might just get up to 200, 4000 people, but them it goes to everyone (Interview; school 3, female)

They have a lot of power to influence people and they don't always necessarily use it positively (Interview; school 8, male)

Official organizations were reported to have the strongest influence on changing young people's health-related behaviours (53%, Online Survey). Young people, however, frequently referred to the influence of celebrities, sports men and women and commercial brands. Sports men and women and commercial brands were viewed as a credible source of information; for example, ‘because they like know what they’re talking about and they’re experts on it, so we could do that’ (Interview; school 2, male). Celebrities, on the other hand, were not considered to be a credible source of information:

I think everyone looks up to these celebrities but we have got to understand they have a certain lifestyle that we are not living. So if they say they are in the gym, they’re not in the gym, they are getting surgery (Interview; school 10, female)

Body transformations, like it's really easy to take photos like five minutes apart that have drastic like differences so quite a lot of them can be fake (Interview; school 8, male)

Despite understanding that celebrities did not provide a credible source of information, young people's health-related understandings and behaviors were influenced by the health-related material celebrities shared. Young people struggled to determine the credibility of information due to the vast quantities of material to which they are exposed and some confusion existed when some of the celebrities posted material that was similar to that posted by the sports men and women.

In our generation, it is just about consumerism, I think. If we see a celebrity with it, we want it. I am not going to lie, I would rather be thicker than what I am now, but that is just because of society (Interview, school 10, female)

I respect the sort of like actors and sports stars but the same issue occurs with them as much as the Kardashians (Interview; school 8, male)

Reputable content was constructed through understandings of credibility, associated with followers and reach. Similar to Giroux’s (2002) reports on how films operate pedagogically, reputable accounts promoted common sense assumptions about health, limited the range of information available to young people and also influenced young people through affect.

## Discussion

This study provides evidence about the ways in which young people understand, experience and generate social media content, and how they report the influence of social media on their health-related knowledge and behaviours. New evidence is provided on: (i) the types of health-related information young people access and attend to; and (ii) contradictions in the ways in which young people attach value to different forms of content and are influenced to attend to it or not. In contrast to much of the risk-related rhetoric and assumptions about dystopian impacts (Frith, 2017; Shaw et al., 2015), the findings reveal that young people respond in different ways to similar health-related social media information. Indeed, it was evident that contrary to popular opinion, many young people were critically aware users and generators of health-related social media. While risks should not be overlooked, and were also apparent in the data, the findings provide clear evidence that social media can also be a powerful educative health resource that has considerable significance in the lives of contemporary young people. At the same time, it was also evident that some young people, some of the time, found themselves in a position of vulnerability either as a result of social media engagement or other issues that became magnified in the social media context. The important point to make is that this a very dynamic environment where young people's physical, social and emotional needs can change rapidly—particularly through adolescence—and negative impacts can escalate quickly as a result of the power of the medium and its content. The challenge for relevant adults who wish to offer support and guidance to young people is to know when young people are in control of social media, and when it shifts into controlling them. There was evidence of both these states in the data.

**Figure 1.**
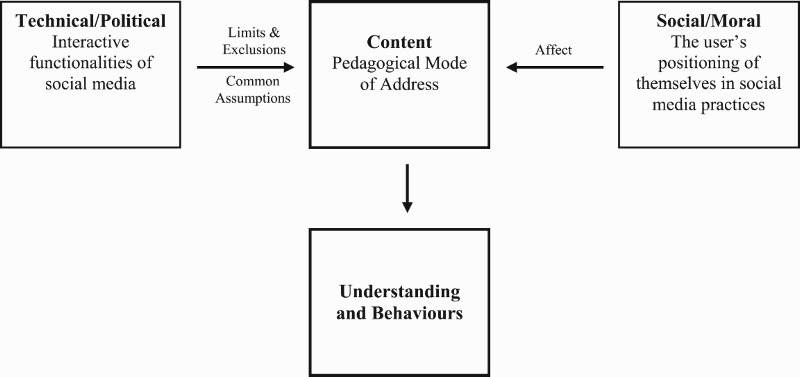
The operation of pedagogy in a social media context.

Public pedagogies initially appeared to provide a neat umbrella concept to capture the informal educational context of social media. Yet the concept, as argued by others, does little to account for young people's diverse experiences, the dynamic interactive environment of social media or the unique operation of pedagogies in a social media context (Andersson & Olson, 2014; Savage, 2014). In response to some of these issues, we extended the public pedagogies analytical framework concept by drawing on anthropological and communicative frameworks. Based on the empirical data generated and the analytical process adopted, we developed an adapted conceptualization of pedagogy in a social media context that places dynamic content at its core (see Figure 1) and that accounts for both diversity and interactivity.

Content, as demonstrated in the data, is constructed at the interplay between the user and their interactions (Miller et al., 2016). Building on the Giroux’s (2004) framing of pedagogy, how content influences understandings and behaviours is underpinned by political and moral practices. Political practices can be largely attributed to the interactive functionalities of social media (Figure 1) and are represented through power, knowledge and ideology (Giroux, 2004). In the data, this technical/political dimension was mainly evident through two of Giroux’s (2002) pedagogical processes: common sense assumptions and the limits and exclusions of information. In our adapted framework, moral practices are associated with the user (Figure 1), specifically highlighting how the young people located themselves in social networks (e.g. friends, followers), social practices (e.g. likes) and discourses (e.g. FitTea). The data showed that this moral/social dimension influenced understandings and behaviours through affect. We suggest, therefore, that the interplay between the user and the interactive functionalities: (i) leads to the construction of content, and (ii) shapes how content influences understandings and behaviours (Figure 1).

The focus on content and the interplay between the user and the interactive functionalities provides an adapted pedagogical and analytical framework that illustrates why young people will respond in different ways to health-related information, and why some young people may be more vulnerable than others. We tentatively refer to this as a ‘dynamic, content-led pedagogical framework’ that recognizes content as a key element, and also the dynamism of content as a unique feature that needs to be accommodated. Importantly, different experiences, social purposes of use, and understandings and emotions will all play a role in shaping how young people engage with health-related social media and how they respond to the power of the medium and content. Placing content at the centre of a pedagogical framework also highlights a challenge to existing concepts of pedagogy and public pedagogy. Formal educational contexts often place learners/learning at the core (Armour & Chambers, 2014; Casey et al., 2017), whereas informal contexts often place emphasis on communication (Andersson & Öhman, 2017) or the platform (Miller et al., 2016). Through a focus on content, our framework bridges conceptualisations of pedagogy focussed on the learner, communication and the platform. The value of a focus on content is furthermore important given that content is an enduring entity in the dynamism of online/digital media (Miller et al., 2016). As technology and media evolve, a dynamic, content-based analytical framework can be applied to diverse technologically-mediated contexts, highlighting the enduring application of this work.

In practice, to realize more of the positive impacts of social media (see Shaw et al., 2015), the data indicate that relevant adults need to be able to support and develop young people's capacity to think critically about the relevance of what they encounter through social media. Giroux (2004) was a strong advocate for connecting informal and formal modes of learning to develop young people's critical thinking skills in relation to the media, and his advocacy is reflected in more recent public pedagogy scholarship (Andersson & Öhman, 2017; Burdick & Sandlin, 2013; Rich & Miah, 2014). Similar to arguments for the use of critical pedagogies in physical education (see Leahy, Burrows, McCuaig, Wright, & Penney, 2016; Powell & Fitzpatrick, 2015) or strengths-based approaches (see McCuaig et al., 2013), Giroux (2004, p. 66) argued that educators should develop ‘context-dependent learning that takes account of student experiences and their relationships to popular culture’. In this sense, the content and how this is shaped by the user and the interactive functionalities (Figure 1) could be used to frame classroom discussions. In keeping with Giroux (2004), however, critical and strengths-based approaches are important ways of framing critical inquiry to ensure that young people can discuss their experiences in ways that do not marginalize their understandings and behaviours (Leahy et al., 2016; McCuaig & Quennerstedt, 2016). In practice, we therefore encourage relevant adults (e.g. teachers, parents/guardians) to develop such context-dependent learning in relation to young people's experiences of social media.

To better equip relevant adults, further research is required on the complex and dynamic relationship between young people, social media and health. This study has made analytical advancements to public pedagogy scholarship through connecting anthropological and communication theory frameworks with public pedagogy scholarship (Figure 1). In turn, an analytical framework that accounts for pedagogy and the varied, multi-dimensional and mass user-generated nature of social media has been proposed. In future work, extended conceptual framings of public pedagogy could be applied. For example, Rich and Miah’s (2014) concept of relationality and public pedagogies could generate understandings of the different contextual and embodied resources young people draw on to construct content. Equally, Andersson and Öhman’s (2017) epistemological move analysis could provide detail on the meaning-making processes to explain why some young people resist content, whereas others do not.

Methodologically, this study has exemplified the importance of understanding young people's perspectives in order to frame how adults can better support young people. The study has reported participatory methods that can be used in future research to generate nuanced understandings of young people's uses of social media. Conceptually, the study was limited in terms of understandings of health and pedagogy. In terms of health, the focus was on physical activity, diet/nutrition and body image. In turn, the methodological questions and the content of class activities may have directed young people towards these particular viewpoints on health (McCuaig & Quennerstedt, 2016). As suggested by McCuaig and Quennerstedt (2016), focussing and framing the research by a focus on what is considered to be a pre-requisite for living a good life could produce different data. In terms of pedagogy, Giroux’s (2002) pedagogical processes provided a useful means to interpret how content influenced understandings and behaviours. There is a need to appreciate, however, that Giroux's work is framed by power and that different pedagogical processes may have existed (Savage, 2014). Methodologically, the sample size was also gender-biased toward females. While gender balance was addressed within interviews and class activities, readings of the online survey data should appreciate a strong focus on females. Ethically, the study was also constrained by interpreting young people's experiences of social media, given that access to young people's social media accounts was rightly restricted. For further rich, robust, and detailed account of young people's experiences further methodological and ethical innovation is required.

## Conclusion

Given the challenges that many relevant adults face in understanding social media, young people currently lack appropriate guidance in their health-related uses of social media. In particular, young people need the kind of support that is responsive to those points in time when young people tip from being in control of the media, to the media controlling them. The findings of this research suggest that relevant adults can support young people to realize more of the positive impacts of social media through an understanding of the importance and essential dynamism of content and how: (i) the interactive functionalities of social media shape what health-related information is accessible to young people, and (ii) how young people's social uses of social media shape the health-related information they attend to.

## Notes

1 For an overview of the characteristics of different types of schools in the UK please see https:// www.gov.uk/types-of-school

2 We’re always working to update the types of photos and videos you see in Search & Explore to better tailor it to you. Posts are selected automatically based on things like the people you follow or the posts you like. You may also see video channels, which can include posts from a mixture of hand-picked and automatically sourced accounts based on topics we think you'll enjoy. (https://help.instagram.com/487224561296752)

3 https:// www.fittea.com

4 There are multiple types of Slim Tea, and a specific brand name has not been provided.
